# Plasma lathosterol measures rates of cholesterol synthesis and efficiency of dietary phytosterols in reducing the plasma cholesterol concentration

**DOI:** 10.1016/j.clinsp.2022.100028

**Published:** 2022-04-06

**Authors:** Valéria Sutti Nunes, Angela de Oliveira Godoy Ilha, Guilherme da Silva Ferreira, Renata de Paula Assis Bombo, Milessa Silva Afonso, Maria Silvia Ferrari Lavrador, Roberta Marcondes Machado, Edna Regina Nakandakare, Eder Carlos Rocha Quintão, Ana Maria Lottenberg

**Affiliations:** a Laboratório de Lipides (LIM10), Hospital das Clínicas, Faculdade de Medicina, Universidade de São Paulo (HCFMUSP), São Paulo, SP, Brazil; b Faculdade Israelita de Ciências da Saúde Albert Einstein (FICSAE), São Paulo, SP, Brazil

**Keywords:** Cholesterol, Lathosterol, Phytosterols, Diet

## Abstract

**Objectives:**

Because the plasma campesterol/cholesterol ratio does not differ between groups that absorb different amounts of cholesterol, the authors investigated whether the plasma Phytosterols (PS) relate to the body's cholesterol synthesis rate measured as non-cholesterol sterol precursors (lathosterol).

**Method:**

The authors studied 38 non-obese volunteers (58±12 years; Low-Density Lipoprotein Cholesterol ‒ LDL-C ≥ 130 mg/dL) randomly assigned to consume 400 mL/day of soy milk (Control phase) or soy milk + PS (1.6 g/day) for four weeks in a double-blind, cross-over study. PS and lathosterol were measured in plasma by gas chromatography coupled to mass spectrophotometry.

**Results:**

PS treatment reduced plasma total cholesterol concentration (-5.5%, *p* < 0.001), LDL-C (-7.6%, *p* < 0.001), triglycerides (-13.6%, *p* < 0.0085), and apolipoprotein B (apo B) (-6.3%, *p* < 0.008), without changing high density lipoprotein cholesterol (HDL-C concentration), but plasma lathosterol, campesterol and sitosterol expressed per plasma cholesterol increased.

**Conclusions:**

The lathosterol-to-cholesterol plasma ratio predicted the plasma cholesterol response to PS feeding. The highest plasma lathosterol concentration during the control phase was associated with a lack of response of plasma cholesterol during the PS treatment period. Consequently, cholesterol synthesis in non-responders to dietary PS being elevated in the control phase indicates these cases resist to further synthesis rise, whereas responders to dietary PS, having in the control phase synthesis values lower than non-responders, expand synthesis on alimentary PS. Responders absorb more PS than non-responders, likely resulting from responders delivering into the intestinal lumen less endogenous cholesterol than non-responders do, thus facilitating greater intestinal absorption of PS shown as increased plasma PS concentration.

## Highlights

• Phytosterols supplementation reduced total cholesterol, LDL-C, apo B, and triglycerides.
• Phytosterols supplementation increased plasma lathosterol as well as campesterol and sitosterol, indicating compliance to the diets.
• Lathosterol-to-cholesterol ratio predicted the cholesterol response to phytosterols feeding.
• High basal cholesterol synthesis is associated with a lack of response to plasma cholesterol.

## Introduction

It is well known that dietary Phytosterols (PS) reduce total plasma cholesterol and low-density lipoproteins cholesterol (LDL-C)^1^, ^2^, ^3^ due to displacement of cholesterol from the intestinal lumen micelles,^4^^,^^5^ and for molecular actions inside enterocytes and hepatocytes.^6^ Moreover, it was also demonstrated that PS could induce LDL receptor expression^7^ and lower plasma endothelin-1 independently of plasma LDL-C reductions contributing to the comprehension of plant sterol's effects on endothelial function and prevention of cardiovascular diseases.^8^ Because of the beneficial effects on lipid profile, the 2001 National Cholesterol Education Program (NCEP ATP-III) (National Cholesterol Education Program Expert Panel) included dietary PS in the treatment for moderate hypercholesterolemia.^9^ However, after this guideline publication, some reports claimed high PS plasma and tissue concentrations related to cardiovascular risk increase.^10^^,^^11^ Nevertheless, Bombo et al.^12^ showed that PS feeding did not accumulate sterols in the aortic valve or arterial wall in LDL receptor knockout mice fed a high saturated fat diet. Furthermore, PS treatment prevented atherosclerotic lesion development in hypercholesterolemia mice models.^12^

The recommendation of PS supplementation to treat hypercholesterolemia is 2 g/day.^13^ Some authors have shown that amounts from 0.8 g/day were effective in reducing cholesterol.^14^ It is not possible to reach the recommended PS intake only with the consumption of vegetable foods, as their habitual diet contains 150‒400 mg/day. A review of approximately 40 studies found that the dose of 2 g/day resulted in a 10% reduction in LDL-C and that larger amounts do not potentiate this action.^15^

Plasma concentrations of PS and of non-cholesterol sterols precursors of cholesterol synthesis, respectively markers of the intestinal cholesterol absorption and of body's cholesterol synthesis have been used as markers of atherosclerotic cardiovascular disease.^16^, ^17^, ^18^, ^19^, ^20^, ^21^ Nonetheless, objections were raised against the interpretation of results utilizing plasma PS measurements as markers of intestinal cholesterol absorption and of non-cholesterol precursors as markers of cholesterol synthesis. In this regard, high plasma PS were considered inappropriate cholesterol absorption surrogates because dietary PS lowered the intestinal cholesterol absorption rate.^22^ Furthermore, in an investigation on moderate human hypercholesterolemia, the plasma campesterol/cholesterol ratio did not differ between groups that absorb different amounts of cholesterol simultaneously measured by the gold standard radioactive or isotopic cholesterol procedure.^23^ Therefore, the elevation of plasma PS may represent a defect in the body's efficiency to re-excrete PS and not an increase in the intestinal absorption of dietary cholesterol.^24^ Consequently, increased PS intestinal uptake relationship to premature atherosclerosis in humans is unlikely. Accordingly, Cardiovascular Disease (CVD) mortality is related reciprocally with plasma PS (sitosterol) as a cholesterol absorption marker, and the high desmosterol/sitosterol ratio suggests high cholesterol synthesis and low absorption associated with high total and CVD mortality.^25^ Nonetheless, low serum lathosterol, but not sterol absorption markers, have been associated with increased CVD.^21^ In contrast, increased excretion of endogenous cholesterol, which represents increased cholesterol synthesis, seems negatively associated with carotid intima-media thickness.^26^ In one study in children, dietary PS altered the serum PS concentration but not concentrations of cholesterol synthesis precursors.^27^ Contrarily, in one study on low cholesterol synthesis cases during the basal period, high intestinal sitosterol absorption occurred on PS feeding,^28^ but this was not mentioned in another study.^29^ Consequently, there are often limitations on the use of serum non-cholesterol sterol synthesis and absorption markers on cardiovascular risk evaluation.^30^

To investigate the reasons for the mentioned published discrepancies, the authors aimed at measuring plasma concentrations of non-cholesterol sterol as a precursor of cholesterol synthesis (lathosterol), and PS as markers of intestinal absorption of cholesterol in the control phase and on the PS feeding phase.

## Materials and methods

### Study design

This study shares previously published casuistic where the authors evaluated the effect of PS on biomarkers involved in atherosclerosis progression and whether these effects are independent of alterations in plasma LDL-C levels.^8^ This is a randomized, double-blind dietary intervention trial lasting 4 weeks each study period. Initially, all the participants were submitted to a 3-week run-in period in which they received the control product (soy milk) to test adherence to the protocol. After the baseline period, participants were randomly assigned to control or to PS treatment phases for 4-weeks; after that, a reverse sequence was immediately carried out. The Control group received 400 mL of soy milk daily; the PS group received 400 mL of soy milk enriched with PS (1.6 g/day) being 78% β-sitosterol-ester, 13% sitostanol-ester, 5.3% campesterol-ester, and 0.5% campestanol-ester. Control and PS soy milk were produced at Unilever Bestfoods Netherlands. The analysis of the composition of the milk was performed by Unilever Bestfoods (Table 1).

**Table 1:** Soy milk nutritional composition per portion (200 mL).

Nutritional composition	Soy milk	Soy milk + PS
Energy (kcal)	138	144
Protein (g)	6.5	6.5
Total fat (g)	4.4	5.0
Polyunsaturated fat	2.3	2.5
Monounsaturated fat	1.0	1.1
Saturated fat	0.7	0.9
Trans fatty acid	0	0
Cholesterol (mg)	0	0
Carbohydrates (g)	18.2	18.2
Total sugar	14.1	14.1
Lactose	0	0
Phytosterol (g)	0	0.8
β-sitosterol-ester	‒	0.63
Sitostanol-ester	‒	0.10
Campesterol-ester	‒	0.05
Campestanol-ester	‒	0.005
Sodium (g)	0.1	0.1

Blood samples for biochemical analysis from fasting participants were drawn on the last day of each period. All participants were advised to maintain their body weight on a normocaloric diet based on the NCEP-ATPIII recommendation: total energy represented as fat (30%), being less than < 10% as saturated fat, and < 300 mg cholesterol/day. They were advised not to consume products enriched with PS during the study. Nutritional monitoring was performed by a registered dietitian using a 24-hour dietary recall to estimate food intake and to ensure diet adherence. Soy milk was supplied weekly on the same day of body weight measurement. Participants were instructed to consume soy milk or PS-enriched soy milk twice daily at lunch and dinner.

Participants (*n* = 38; female 31 and male 7), aged 38‒77 years, were recruited in the Dyslipidemia Outpatient Unit of the Endocrinology and Metabolism Service and staff members of the Hospital das Clinicas, Faculdade de Medicina, Universidade de São Paulo, and participated in body weight and height screenings. Blood samples were drawn for the lipid profile determination. Inclusion criteria were Body Mass Index (BMI) between 20 and 30 kg/m^2^, total cholesterol between 200‒300 mg/dL, LDL-C ≥ 130 mg/dL, and triglycerides ≤ 250 mg/dL (Table 2). Exclusion criteria were obesity, use of lipid-lowering medication or a prescribed diet in the previous month, alcohol abuse or illicit drug users, pregnancy or breastfeeding, smoking, diabetes mellitus, hypothyroidism, renal or hepatic diseases, or participation in another lifestyle or pharmaceutical intervention study. All subjects provided informed written consent. The Ethics in Research Committee of the Hospital das Clinicas, Faculdade de Medicina, Universidade de São Paulo, approved the study protocol (CAPPesq n° 112/06). All methods were in accordance with the approved guidelines and in agreement with the Ethical Principles for Medical Research Involving Human Subjects as stated by the Declaration of Helsinki.

**Table 2:** Subjects characteristics at baseline.

Parameter	Mean ± SD
n	38
Age (years)	58 ± 12
Weight (Kg)	64 ± 10
BMI (kg/m^2^)	25.3 ± 2.4
Total cholesterol (mg/dL)	245 ± 34
Triglycerides (mg/dL)	141 ± 53
LDL-C (mg/dL)	165 ± 34
HDL-C (mg/dL)	49 ± 12

BMI, Body Mass Index; LDL-C, Low-Density Lipoprotein Cholesterol; HDL-C, High Density Lipoprotein Cholesterol.

### Lipid profile

After fasting for 12 hours, blood samples were transferred into tubes containing Ethylenediamine Tetraacetic Acid (EDTA). Plasma was immediately separated by centrifugation and the following preservatives were added: 0.25% chloramphenicol plus 0.5% gentamycin (20 μL/mL), 2 mmoL benzamidine/L (5 μL/mL), 10 mmoL phenyl-methyl-sulfonyl fluoride/L (0.5 μL/mL), and aprotinin (0.5 μL/mL).

Plasma lipid concentrations (total cholesterol, HDL-C, and triglycerides) were measured enzymatically using a COBAS MIRA (Roche Diagnostics, Basle/Basel, Switzerland), using kits from Roche Diagnostics (Mannheim, Germany). HDL-C was measured after apolipoprotein (apo) B-containing lipoprotein precipitation^31^ by dextran sulfate and magnesium chloride 2 M (1:1) (50 µL/500 µL of plasma) solution. The LDL-C was calculated according to the Friedewald formula,^32^ and apo B was measured by the turbidimetric method (Randox Laboratories, United Kingdon).

### Sterols analyses

Plasma lathosterol, campesterol, and sitosterol were measured by Gas Chromatography (GC) coupled to a Mass Spectrophotometer (MS) (Shimadzu GCMS-QP2010 Plus, Kyoto, Japan), with the software GCMS solution version 2.5.^33^, ^34^, ^35^ Plasma samples (100 μL) added 5α-cholestane (1 μg) as the internal standard were hydrolyzed with KOH in ethanol (1 moL/L, 1 mL) at 60°C (1h) and extracted with hexane. Sterols were derivatized with a silylating solution of pyridine and BSTFA (N, O-bis [trimethylsilyl] trifluoroacetamide) +1% TMCS (trimethylchlorosilane) (1:1, v/v) (Supelco 33155-U) for 1h at 60°C. The derivatized sample (1 µL) was injected into a gas chromatograph coupled to a mass spectrometer (Shimadzu GCMS-QP2010, Kyoto, Japan). Efficient sterol separation was achieved in a Restek capillary column (100% dimethyl polysiloxane ‒ Rxi13323) that was 30m long, had a 0.25 mm internal diameter, contained helium as the mobile phase, and had a constant linear velocity of 45.8 cm/s with an oven temperature at 280°C. The mass spectrometer was operated in electron impact mode at an ionization voltage of 70 eV with a source temperature of 300°C for the ions and the interface. Single Ion Monitoring (SIM) was carried out by monitoring m/z = 109, 149 and 217 for 5α-cholestane, m/z = 213, 255 and 458 for lathosterol, m/z = 129, 343 and 382 for campesterol and m/z = 129, 357 and 396 for sitosterol enabling greater sensitivity in quantification. Quantification was based on the Total Ion Chromatogram (TIC) with correction by the internal standard 5α-cholestane, and identification was based on comparison with the retention times and mass spectra of the standard curve.^36^ The coefficient of variation of the method was: lathosterol 5%, campesterol 6%, and sitosterol 7%.

### Statistical analysis

Comparisons between the Control and PS groups were by paired Student's *t*-test. The influence of the degree of hypercholesterolemia over the PS response and PS response patterns related to LDL-C was by unpaired Student's *t*-test. Data are shown as means and standard deviation. Analyses were performed utilizing the GraphPad Prisma version 4.00, and the significance level was considered as *p* < 0.05.

## Results and discussion

### Plasma sterol concentrations

Participants' (*n* = 38) body weight and BMI remained unaltered throughout the study (Table 3). PS reduced total cholesterol, LDL-C, apoB and triglycerides without affecting HDL-C plasma concentrations. PS supplementation increased plasma level (µg/mL) and ratios (µg/mg cholesterol) of campesterol and sitosterol indicating the participant's compliance to diets. Plasma lathosterol level (µg/mL) did not change while lathosterol ratios (µg/mg cholesterol) increased due to blockade of intestinal cholesterol absorption by PS. However, the lathosterol/phytosterols ratios decreased due to the predominant PS absorption increase.

**Table 3:** Body weight, BMI, biochemical analysis, plasma sterol concentrations of patients in the Control and on the PS phases, *n* = 38.

	Control	PS	p
**Body weight (kg)**	64.9 ± 10.2	65.1 ± 10.3	0.08
**BMI (kg/m^2^)**	25.4 ± 0.4	25.4 ± 0.4	ns
**Total cholesterol (mg/dL)**	261 ± 7.1	244 ± 5.8	<0.001
**HDL-C (mg/dL)**	46 ± 1.7	48 ± 1.9	ns
**LDL-C (mg/dL)**	183 ± 5.9	169 ±5.2	0.001
**ApoB (mg/dL)**	126 ±3.7	118 ± 3.2	0.006
**Triglycerides (mg/dL)**	154 ± 10	133 ± 7	0.008
**Plasma sterols expressed as µg/mL**			
Lathosterol	4.07±1.27	4.07±0.76	ns
Campesterol	4.95±1.47	5.64±1.47	<0.001
Sitosterol	4.15±1.24	4.85±1.21	<0.001
**Plasma sterols expressed as µg/mg cholesterol**			
Lathosterol	1.53 ± 0.09	1.69 ± 0.06	0.012
Campesterol	1.96 ± 0.12	2.34 ± 0.11	0.02
Sitosterol	1.64 ± 0.09	2.02 ± 0.09	<0.001
Lathosterol/Campesterol ratio	0.85 ± 0.05	0.76 ± 0.03	<0.001
Lathosterol/Sitosterol ratio	1.03 ± 0.05	0.88 ± 0.04	<0.001

BMI, Body Mass Index; LDL-C, Low-Density Lipoprotein Cholesterol; HDL-C, High Density Lipoprotein Cholesterol; apoB, apolipoprotein B. Data shown as means and standard deviation. Student's *t*-test.

The consumption of PS-enriched soy milk significantly lowered total cholesterol (-5.5%) and LDL-C (-7.6%) (Table 3). This very mild cholesterol reduction could be attributed to less PS intake in this study as compared to other investigations.^12^^,^^37^ Nonetheless, blood cholesterol variation allowed the identification of different patterns of metabolic changes elicited by PS feeding agreeing with the wide variability in individual LDL-C plasma reduction response to PS intake previously reported.^1^ As compared to the Control phase, PS reduced apoB-LP likely belonging to LDL, but increased TG plasma concentrations, especially in participants presenting higher LDL-C concentrations at baseline, possibly because in the latter hepatic VLDL-C synthesis is high.^38^

### LDL-C tertiles selected at baseline

In order to investigate whether the degree of hypercholesterolemia influences the PS response, patients were divided according to tertiles of plasma LDL-C identified at baseline (< 168 mg/dL and > 187 mg/dL) (Table 4). Data variations on the Control phase minus the PS phase are shown as delta values. PS intake effectively reduced LDL-C and apo B concentrations in both phases but failed to modify triglycerides concentrations. Furthermore, plasma concentrations of lathosterol, campesterol, and sitosterol properly corrected for plasma cholesterol were higher in the LDL-C < 168 than in LDL-C > 187 tertiles during the control phase and failed to change on PS feeding. These results preliminarily indicate similar behavior of the synthesis and absorption markers in the groups that differ by LDL-C concentration.

**Table 4:** Patients' data during study periods according to the averages of LDL-C tertiles selected at baseline (< 168 mg/dL and > 187 mg/dL).

	Study periods	LDL-C < 168	LDL-C > 187	p
LDL-C (mg/dL)	Control	150 ± 14 (*n* = 13)	215± 20 (*n* = 12)	<0.0001
	PS	148 ± 15 (*n* = 13)	192 ± 24 (*n* = 12)	<0.0001
Delta LDL-C (%)		-0.5 ± 13	-10.3 ± 12	ns
Delta LDL-C (mg)		-1.9 ± 18.1	-23.0 ± 26.1	0.0269
ApoB (mg/dL)	Control	110 ± 10 (*n* = 13)	151 ± 22 (*n* = 13)	<0.0001
	PS	112 ± 14 (*n* = 13)	131 ± 22 (*n* = 13)	0.0120
Delta ApoB (%)		1.8 ± 10.1	-12.5 ± 10.2	0.0019
Delta ApoB (mg)		1.8 ± 11.5	-19.3 ± 17.5	0.0013
Triglycerides (mg/dL)	Control	96 ± 7 (*n* = 13)	130 ± 14 (*n* = 13)	<0.0001
	PS	115 ± 38 (*n* = 13)	148 ± 44 (*n* = 13)	0.0472
Delta triglycerides (%)		22 ± 36	15 ± 38	ns
Delta triglycerides (mg)		19 ± 42	18 ± 47	ns
Lathosterol (µg/mg cholesterol)	Control	1.689 ± 0.314 (*n* = 11)	1.372 ± 0.303 (*n* = 12)	0.0221
	PS	1.887 ± 0.250 (*n* = 11)	1.522 ± 0.233 (*n* = 12)	0.0016
Delta lathosterol (%)		13.73 ± 15.42	14.15 ± 22.77	ns
Delta lathosterol (µg/mg)		0.197 ± 0.214	0.150 ± 0.286	ns
Campesterol (µg/mg cholesterol)	Control	2.370 ± 0.633 (*n* = 11)	1.391 ± 0.345 (*n* = 12)	0.0002
	PS	2.881 ± 0.754 (n=11)	1.941 ± 0.518 (*n* = 11)	0.0028
Delta campesterol (%)		25.43 ± 32.30	40.71 ± 20.73	ns
Delta campesterol (µg/mg)		0.511 ± 0.704	0.550 ± 0.280	ns
Sitosterol (µg/mg cholesterol)	Control	1.952 ± 0.445 (*n* = 11)	1.196 ± 0.283 (*n* = 11)	0.0001
	PS	2.484 ± 0.518 (*n* = 11)	1.693 ± 0.451 (*n* = 11)	0.0011
Delta sitosterol (%)		30.16 ± 26.87	41.91 ± 17.95	ns
Delta sitosterol (µg/mg)		0.532 ± 0.506	0.497 ± 0.242	ns

LDL, Low-Density Lipoprotein; apoB, apolipoprotein B. Control minus phytosterol data variations are expressed as delta values. Data shown as means and standard deviation. Unpaired Student's *t*-test.

The similarity of plasma concentrations of lathosterol and PS in the LDL-C < 168 and LDL-C > 187 groups (Table 4) suggests that several factors participate simultaneously in hypercholesterolemia such as variations in synthesis, absorption, and retention of sterols in plasma being often impossible to distinguish the participation of each one of them. This is an example that there are technical limitations on the usefulness of these markers, as previously indicated.^23^^,^^30^ Nonetheless, the present investigation confirms previous studies showing that the serum lathosterol-to-cholesterol ratio predicts the serum cholesterol responsiveness on PS feeding. However, during placebo, lack of plasma cholesterol response to PS occurs when the cholesterol synthesis rate is high.^28^^,^^29^ The present report differs from the study by Mackay DS et al.^28^ because the latter investigated obese compared to non-obese while the present study excluded obese participants. Obesity increases cholesterol synthesis.^20^^,^^39^ Furthermore, these results contradict a study in children in which dietary PS altered the concentration of serum PS, but not the serum concentration of cholesterol synthesis precursors.^27^ It is possible that in children, the effect of PS on blood cholesterol differs from adults because cholesterol synthesis often is higher in children than in adults.^30^

### Non-responders and responders to PS treatment

The authors also examined whether plasma sterol response patterns defined by LDL-C changes distinguish patients' non-responders (*n* = 10) and responders (*n* = 27) to PS treatment (Table 5). Control phase minus PS phase data variations is expressed as delta values. The authors found in the Control phase lathosterol higher in non-responders than in responders to PS. However, on PS feeding, concentrations of lathosterol did not differ between the two groups (non-responders vs. responders). On the other hand, lathosterol percent variation on PS in relation to the Control phase did not vary in non-responders and increased in responders. This means that non-responders, because of high synthesis before PS treatment, could not further expand synthesis on PS treatment. Responders synthesize less in the Control phase but expand the synthesis rate on PS.

**Table 5:** Plasma sterol response patterns defined by LDL-C changes distinguish patients' non-responders and responders to PS treatment.

	Study periods	Non-responders (*n* = 11)	Responders (*n* = 27)	p
LDL-C (mg/dL)	Control	171 ± 24	191 ± 37	ns
	PS	184 ± 29	165 ± 32	ns
Delta LDL-C (%)		8 ± 5	-13 ± 7	<0.0001
Delta LDL-C (mg/dL)		14 ± 9	-26 ± 15	<0.0001
ApoB (mg/dL)	Control	122 ± 24	129 ± 23	ns
	PS	124 ± 21	117 ± 19	ns
Delta ApoB (%)		3 ± 12	-9 ± 11	0.0044
Delta apoB (mg/dL)		3 ± 14	-12 ± 16	0.0039
Triglycerides (mg/dL)	Control	110 ± 16	113 ± 18	ns
	PS	139 ± 50	132 ± 41	ns
Delta Triglycerides (%)		26 ± 43	19 ± 44	ns
Delta Triglycerides (mg/dL)		28 ± 49	19 ± 45	ns
Lathosterol (µg/mg cholesterol)	Control	1.929 ± 0.954	1.463 ± 0.363	0.0379
	PS	1.658 ± 0.411	1.686 ± 0.344	ns
Delta lathosterol (%)		-8 ± 16	18 ± 19	0.0009
Campesterol (µg/mg cholesterol)	Control	2.242 ± 0.898	1.831 ± 0.610	ns
	PS	2.168 ± 0.582	2.401 ± 0.755	ns
Delta campesterol (%)		3 ± 25	34 ± 26	0.0053
Sitosterol (µg/mg cholesterol)	Control	1.958 ± 0.860	1.529 ± 0.469	ns
	PS	1.841 ± 0.532	2.075 ± 0.595	ns
Delta sitosterol (%)		1 ± 26	38 ± 23	0.0007

LDL, Low-Density Lpoprotein. Control minus phytosterol data variations are expressed as delta values. Data shown as means and standard deviation. Unpaired Student's *t*-test.

Since the degree of cholesterol absorption indicated by plasma PS concentration could influence cholesterol synthesis in the Control phase and its response to PS intake, the authors measured plasma PS concentrations before and after PS feeding. The authors noted in the control phase that plasma absorption markers did not differ between responders and non-responders, but lathosterol was higher in non-responders to PS feeding (Table 5). However, unlike non-responders, responders increased the absorption of PS identified by increased plasma PS concentration, most likely due to a small intestinal lumen cholesterol content competing for intestinal absorption with alimentary PS. The authors suggest that elevated synthesis during Control in non-responders makes them resistant to further synthesis rise on PS treatment, whereas responders can expand synthesis under the effect of alimentary PS because they have lower rates of synthesis than non-responders in the Control phase.

Interestingly, in the Control phase, as well as on PS, plasma concentrations of campesterol and sitosterol did not differ between non-responders and responders. However, as occurred for lathosterol, the percent variation of these markers of absorption on PS feeding over the Control phase was significantly greater in the responders than in the non-responders. This is compatible with the responders absorbing more PS than non-responders.

## Conclusion

The present study's data explain decreased intestinal absorption of cholesterol in metabolic syndrome associated with diminished efficiency of food PS esters in reducing blood cholesterol, although the cholesterol synthesis markers were not measured ^40^. Such a result may be consequent to elevated cholesterol synthesis in metabolic syndrome ^20^.

In summary, responders absorb more PS than non-responders, likely resulting from responders delivering less endogenous cholesterol into the intestinal lumen. The existence of cases responsive to phytosterols fully justifies its use as a food additive, but certain genetic influences on the type of response need investigation. Limitations in the use of blood sterols as markers of cholesterol synthesis and absorption to some extent may have influenced the interpretation of the results.

## Authors’ contributions

**Nunes VS:** Conceptualization, Methodology, Investigation, Writing - Review & Editing, **Ilha AOG;** Methodology, Investigation, Writing - Original Draft, **Lottenberg AM:** Conceptualization, Supervision, Writing - Review & Editing, **Ferreira GS:** Formal analysis, **Bombo RPA:** Investigation, **Afonso MS:** Investigation, **Lavrador MSF:** Investigation, **Machado RM:** Investigation, **Nakandakare ER:** Formal analysis, Validation, **Quintão ECR:** Conceptualization, Writing - Review & Editing.

## Conflicts of interest

The authors declare no conflicts of interest.
